# Etymologia:* Francisella tularensis*

**DOI:** 10.3201/eid1705.ET1705

**Published:** 2011-05

**Authors:** Nancy Männikkö

**Affiliations:** Author affiliation: Centers for Disease Control and Prevention, Atlanta, GA, USA

**Keywords:** etymologia, francisella tularensis

## [fran-sĭ-sel′ə too′′lə-ren-sis]

While studying plague in ground squirrels in 1911, George McCoy and Charles Chapin discovered a bacterium that caused a different disease. They named the pathogen *Bacterium tularense *after Tulare County, California, location of their study. In 1928, Edward Francis, a US Public Health Service bacteriologist, linked *B. tularense *with deer fly fever―tularemia transmitted by deer flies from infected wild rabbits to humans. In 1974, *B. tularense *was renamed *Francisella tularensis* in recognition of Dr. Francis’ many contributions to our knowledge of tularemia.

Sources: Barry, J. Notable contributions to medical research by public health scientists. Public Health Service Publication No. 752. 1960 [cited 2011 Feb 25]. http://history.nih.gov/research/downloads/Notable_Cont_Med_Research.pdf; Francis E. Sources of infection and seasonal incidence of tularemia in man. Public Health Rep. 1937;52:103–13; McCoy GW, Chapin CW. Further observations on a plague-like disease of rodents with a preliminary note on the causative agent, *Bacterium tularensis*. J Infect Dis. 1912;10:61–72; Sjöstedt A. Tularemia: history, epidemiology, pathogen physiology, and clinical manifestations. Ann N Y Acad Sci. 2007;1105:1–29. PubMed doi:10.1196/annals.1409.009

